# Editorial: HFpEF and HFmrEF: Different Sides of the Same Coin?

**DOI:** 10.3389/fcvm.2022.916534

**Published:** 2022-05-05

**Authors:** Manuel Martínez-Sellés, Dachun Xu, Jian Zhang, Sang-Bing Ong

**Affiliations:** ^1^Cardiology Department, Hospital General Universitario Gregorio Marañón, CIBERCV, Universidad Europea, Universidad Complutense, Madrid, Spain; ^2^Department of Cardiology, Shanghai Tenth People's Hospital, Tongji University School of Medicine, Shanghai, China; ^3^Fuwai Hospital, Chinese Academy of Medical Sciences and Peking Union Medical College, Beijing, China; ^4^Department of Medicine and Therapeutics, Faculty of Medicine, Chinese University of Hong Kong (CUHK), Shatin, Hong Kong SAR, China; ^5^Centre for Cardiovascular Genomics and Medicine (CCGM), Lui Che Woo Institute of Innovative Medicine, CUHK, Shatin, Hong Kong SAR, China; ^6^Hong Kong Hub of Paediatric Excellence (HK HOPE), Hong Kong Children's Hospital (HKCH), Kowloon Bay, Hong Kong SAR, China; ^7^Kunming Institute of Zoology - The Chinese University of Hong Kong (KIZ-CUHK) Joint Laboratory of Bioresources and Molecular Research of Common Diseases, Kunming Institute of Zoology, Chinese Academy of Sciences, Kunming, China

**Keywords:** heart failure, elderly, ejection fraction, prognosis, pathophysiology

Heart failure (HF) has traditionally been divided into distinct phenotypes based on left ventricular ejection fraction (LVEF). The most common way to evaluate LVEF is echocardiography, yet its measurements are subject to substantial variability associated with the technique itself as well as hemodynamic conditions of the patient. In any case, as clinical trials have used specific cut-offs for LVEF, some treatment benefits have only been proven below a certain LVEF value. This is the main reason that explains the recommendation of the European Society of Cardiology in the use of the following three categories (1): HF with reduced ejection fraction (HFrEF, LVEF ≤ 40%), HF with mildly reduced ejection fraction (HFmrEF, LVEF 41–49%), and HF with preserved ejection fraction (HFpEF, LVEF>50%). In any case, most studies that have included patients with HFmrEF suggest that they may benefit from similar therapies to those with HFrEF. This was the main reason for the recent change of the name in the group of patients with LVEF 41–49% that was previously named “heart failure with mid-range ejection fraction.”

This Research Topic aims to focus on patients with HFpEF and HFmrEF, highlighting their similarities and differences. The clinical profile of these patients has particularities that differentiate them from HFrEF, including a more advanced age and a higher prevalence in women (2, 3). In addition, biomarkers and ionic parameters have also a different impact according to LVEF and their role, levels and thresholds in HFpEF and HFmrEF are different from the ones observed in HFrEF (4).

## HFpEF

In this special volume, Chi et al. review the role of arterial stiffness and its current treatment strategies. Several original clinical studies are also presented. Bai et al. evaluate the interrelation between neutrophil to lymphocyte ratio and diastolic dysfunction, showing that a high neutrophil to lymphocyte ratio coupled with transcriptional activation of neutrophils correlates with systemic inflammation and functional impairment. Liang et al. present a *post-hoc* analysis of the Treatment of Preserved Cardiac Function Heart Failure with an Aldosterone Antagonist Trial (TOPCAT) focusing on liver function. The authors found that elevated serum cholestasis markers such as total bilirubin and alkaline phosphatase were associated with a poor clinical outcome. Wang et al. show that the MELD-XI score is associated with short-term adverse events in these patients and provides additional discriminatory capacity to risk stratification models in hospitalized patients. Huang et al. describe the association of weight change with mortality risk in patients from the Americas from the Treatment of Preserved Cardiac Function Heart Failure with an Aldosterone Antagonist study, showing that weight loss is related with all-cause mortality, while weight gain is not associated with better survival.

Animal studies are also presented whereby, Zhang et al. describe the alteration of N6-methyladenosine RNA methylation in patients and in a mouse model of HFpEF, suggesting that the modulation of epitranscriptomic processes might be an interesting target for therapeutic interventions. Zhao W. et al. demonstrate how cardiomyocyte-specific deletion of STAT3 results in a murine HFpEF model, an interesting model that could help us to better understand this condition and to test new therapies.

## HFmrEF

With regards to HFmrEF. Zhu et al. summarize the current knowledge regarding clinical epidemiology, pathophysiology, and prognosis of HFmrEF. Ma T. et al. review the treatment regime, showing data that support a similar approach to HFrEF. Palazzuoli and Beltrami review the (few) differences of HFmrEF and HFpEF and emphasize that a same patient evaluated in different periods or by different physicians could lead to varying classification from HFmrEF to HFpEF. Zhou et al. suggest that HFmrEF may represent a transitional stage. Maeder et al. describe the important role of pulmonary hypertension in mediating HFmrEF.

## HF Pathophysiology

Two reviews from this Research Topic focus on HF pathophysiology. Zhao Y-L. et al. perform a systematic review and meta-analysis to compare the effectiveness of exercise training for patients with chronic thromboembolic pulmonary hypertension after pulmonary endarterectomy, concluding that exercise training may be associated with a significant improvement in the exercise capacity and quality of life. Bingel et al. describe the hemodynamic changes during physiological and pharmacological stress testing in HF patients presenting reference values that can help to estimate the expected hemodynamic responses.

Several original studies report interesting finding on HF patients. Qin et al. demonstrate how epicardial adipose tissue measured from computed tomography predicts cardiac resynchronization therapy response in patients with non-ischemic HFrEF. Ma Z. et al. describe a new biomarker, elabela, and show how low plasma levels in hypertensive HF may predict the occurrence of major adverse cardiac events. Pang et al. demonstrate how TRAF family member associated NF-κB accelerates the progression of pathological cardiac hypertrophy and is a potential therapeutic target. Ma M. et al. use a single-cell transcriptome analysis to decipher new potential regulation mechanism of angiotensin-converting enzyme 2 and NPs signaling among HF patients infected with SARS-CoV-2, suggesting that in the failing heart, the upregulation of ACE2 and virus-associated genes could potentially facilitate SARS-CoV-2 virus entry and replication in vulnerable cardiomyocytes. Weijing et al. present the results of a randomized trial showing how cardiac shock wave therapy can ameliorate myocardial ischemia in patients with chronic refractory angina pectoris, an important finding as ischemic heart disease is a common cause of HF.

In summary, this Research Topic highlights the importance of distinguishing between HFpEF and HFmrEF. The prevalence of HF with LVEF <40% is similar or even higher than the prevalence of HFrEF, but the amount of data regarding these conditions is quite scarce when compared against the number of clinical trials that have shown important benefits of HFrEF treatments. Further studies specifically focused on these patients may help to clarify their pathophysiology and to provide new therapeutic tools.

## Author Contributions

MM-S designed the manuscript and wrote the first draft. All authors contributed and approved the final draft.

## Conflict of Interest

The authors declare that the research was conducted in the absence of any commercial or financial relationships that could be construed as a potential conflict of interest.

## Publisher's Note

All claims expressed in this article are solely those of the authors and do not necessarily represent those of their affiliated organizations, or those of the publisher, the editors and the reviewers. Any product that may be evaluated in this article, or claim that may be made by its manufacturer, is not guaranteed or endorsed by the publisher.

## References

[1] Authors/Task Force Members McDonagh TA Metra M Adamo M Gardner RS Baumbach A. 2021 ESC Guidelines for the diagnosis and treatment of acute and chronic heart failure: developed by the Task Force for the diagnosis and treatment of acute and chronic heart failure of the European Society of Cardiology (ESC). With the special contribution of the Heart Failure Association (HFA) of the ESC. Eur J Heart Fail. (2022) 24:4–131. 10.1002/ejhf.2333 35083827

[2] Martínez-Sellés M Ayesta A Díaz-Molina B Bayes-Genis A Baranchuk A. Editorial: the role of sex in heart failure and transplantation. Front Cardiovasc Med. (2021) 8:690438. 10.3389/fcvm.2021.690438 34113666 PMC8185200

[3] Postigo A Martínez-Sellés M. Sex influence on heart failure prognosis. Front Cardiovasc Med. (2020) 7:616273. 10.3389/fcvm.2020.616273 33409293 PMC7779486

[4] Vicent L Alvarez-Garcia J Gonzalez-Juanatey JR Rivera M Segovia J Worner F. Prognostic impact of hyponatraemia and hypernatraemia at admission and discharge in heart failure patients with preserved, mid-range and reduced ejection fraction. Intern Med J. (2021) 51:930–8. 10.1111/imj.14836 32237007

